# The effects of face mask on speech production and its implication for forensic speaker identification-A cross-linguistic study

**DOI:** 10.1371/journal.pone.0283724

**Published:** 2023-03-30

**Authors:** Puyang Geng, Qimeng Lu, Hong Guo, Jinhua Zeng

**Affiliations:** Department of Audio, Video, and Electronic Forensics, Academy of Forensic Science, Shanghai, China; University of Auckland, NEW ZEALAND

## Abstract

This study aims to understand the effects of face mask on speech production between Mandarin Chinese and English, and on the automatic classification of mask/no mask speech and individual speakers. A cross-linguistic study on mask speech between Mandarin Chinese and English was then conducted. Continuous speech of the phonetically balanced texts in both Chinese and English versions were recorded from thirty native speakers of Mandarin Chinese (i.e., 15 males and 15 females) with and without wearing a surgical mask. The results of acoustic analyses showed that mask speech exhibited higher F0, intensity, HNR, and lower jitter and shimmer than no mask speech for Mandarin Chinese, whereas higher HNR and lower jitter and shimmer were observed for English mask speech. The results of classification analyses showed that, based on the four supervised learning algorithms (i.e., Linear Discriminant Analysis, Naïve Bayes Classifier, Random Forest, and Support Vector Machine), undesirable performances (i.e., lower than 50%) in classifying the speech with and without a face mask, and highly-variable accuracies (i.e., ranging from 40% to 89.2%) in identifying individual speakers were achieved. These findings imply that the speakers tend to conduct acoustic adjustments to improve their speech intelligibility when wearing surgical mask. However, a cross-linguistic difference in speech strategies to compensate for intelligibility was observed that Mandarin speech was produced with higher F0, intensity, and HNR, while English was produced with higher HNR. Besides, the highly-variable accuracies of speaker identification might suggest that surgical mask would impact the general performance of the accuracy of automatic speaker recognition. In general, therefore, it seems wearing a surgical mask would impact both acoustic-phonetic and automatic speaker recognition approaches to some extent, thus suggesting particular cautions in the real-case practice of forensic speaker identification.

## 1. Introduction

Coronavirus Disease 2019 (COVID-19) is a transmissible respiratory disease that is highly contagious from person to person. Face mask, as a non-pharmacological intervention imposed by the ongoing global COVID-19 pandemic, has been used to contain the transmission of the disease. Although wearing face mask is no longer a mandatory provision in some countries (e.g., USA, Singapore), it is still a recommended way to prevent the fast spreading of COVID-19.

It has been claimed that face mask, as a form of a low-pass filter, would show distinct effects on speech production in terms of attenuation, resonant peak, etc. [1, 2]. Therefore, along with the ongoing wide use of face mask, acoustic research on speech with face mask has engrossed a lot of attention from the field of phonetics.

### 1.1 Acoustic changes in speech with face mask

Researchers have been continuously engaged in studies on the effect of face mask on speech production across various languages. However, a consensus has not been reached.

Some scholars have conducted acoustic analyses on a sustained vowel (i.e., /a/) produced with and without face mask. Cavallaro et al. collected recordings from 50 speakers (20 men) and detected no acoustic differences (i.e., fundamental frequency, jitter, shimmer, and harmonics-to-noise ratio) between the two scenarios of wearing a surgical mask and no surgical mask [3]. Later, this research team further expanded the amounts of participants (i.e., 60 speakers) and reported similar findings that the effect of surgical mask was not significant on sustained vowel production [4]. These results are consistent with those of Gojayev et al. and Joshi et al. who found no differences in terms of fundamental frequency (henceforth F0), jitter, shimmer, phonation time, harmonic-noise-ration (i.e., HNR), and vowel formants (e.g., F1, F2) with and without a surgical mask [5, 6]. However, a study conducted on Mandarin Chinese speakers showed inconsistent results. Lin et al. collected recordings of a sustained /a/ from 53 speakers (25 men) and found a significantly higher sound pressure level, a smaller perturbation (i.e., decreased jitter and shimmer), and an evident decrease in F3 after wearing a medical mask [7].

As for other studies, it seems that more significant effects of face mask were observed when the acoustic analyses were conducted on continuous speech rather than a solely sustained vowel. Magee et al. collected recordings from seven (near-)native English speakers (4 men; 2 near-native speakers were subsequent bilinguals who had been exposed to English for 15 years and 26 years, respectively). They found that the power distribution in frequencies, measures of timing, and spectral tilt were significantly impacted by wearing N95, while cepstral and harmonics to noise ratios remained unchanged across mask types (i.e., N95, surgical, and cloth masks) [8]. In the same vein, Nguyen et al. found significant attenuation of mean spectral level in 1–8 kHz region and no significant change at 0–1 kHz with face mask in connected speech [9]. To get down to a more detailed analysis, Nguyen and his colleagues particularly investigated the acoustic features of four fricatives (i.e., /f/, /s/, /ʃ/, and /z/) produced by 16 speakers (4 men). They found a significantly lower amplitude of root mean square and center of gravity of /f/ in surgical and N95 masks compared with non-mask conditions [10]. Similar effects were also observed for 10 English speakers (5 men) that face cover equipment (e.g., surgical mask, helmet, hoodie, etc.) would attenuate the overall intensity of sibilants (i.e., /s/ and /ʃ/) [11]; and for 50 Flemish Dutch speakers (21 men) that almost all acoustic features (e.g., median F0, median intensity, jitter, shimmer, smoothed cepstral peak prominence, and formant related-measures) exhibited significant changes in a VESPA sound-playing setup (a head model simulated a speaker wearing a face mask) [12]. For other devices used to prevent disease transmission, Gojayev et al. found a lower shimmer and a higher HNR for a valved face-filtering piece-3 (FFP3) as compared to the values measured with and without a surgical mask [5]. Some scholars have also identified the effect of speaking styles on masked speech production. For example, Knowles and Badh have claimed that the overall acoustic patterns of speech wearing face mask are consistent across three speaking styles (i.e., loud speech, clear speech, and habitual speech) [13].

Some scholars have suggested that those acoustic variations of mask speech might result from the speakers’ adjustments to improve their speech intelligibility and comprehensibility [14, 15]. For example, it has been argued that speaking loudly (e.g., higher intensity), clearly (e.g., higher HNR), and slowly (e.g., slower speech rate) were common speech strategies of the speakers to compensate intelligibility when wearing face mask [8, 15]. Moreover, other study also suggested that increased vocal fatigue during mask speech production might result in other compensatory changes, such as an enlarged vowel space [16].

### 1.2 Recognition of speech with face mask

Due to the effects of face mask on speech production, it seems reasonable to presume that face mask would influence speech recognition. For human listeners, some scholars have found that surgical mask show little effect on speech recognition [17–19], while other scholars have found that cloth mask and N95 mask would affect the recognition accuracy [17, 20]. Toscano and Toscano investigated the effects of four face masks (a surgical mask, an N95 mask, and two cloth masks) on the recognition of spoken sentences in multi-talker babble noise. They found that, in low levels of background noise, masks had little to no effect, with no more than a 5.5% decrease in mean accuracy compared to a no-mask condition. In high levels of noise, mean accuracy was 2.8–18.2% lower than the no-mask condition, but the surgical mask continued to show no significant difference [21].

Besides, it has been reported that listeners with hearing impairment exhibit greater difficulties recognizing masked speech [22–24]. Some have also claimed that, as compared to auditory-only cues, visual cues (e.g., transparent mask) would improve the perceptual accuracy of masked speech, especially when face mask is combined with the presence of background noise [25]. Truong and Weber report similar findings that, for speech produced with a surgical mask, visual cues enhance not only the listener’s intelligibility but also their cued-recall performance [26]. However, Brown et al. suggest that transparent mask would not improve the intelligibility of masked speech as compared to surgical and N95 masks [17].

Furthermore, few studies on speech with face mask have been conducted from the perspective of automatic speech recognition. Early study has claimed that the average accuracies of speaker identification were above 95% across the four face cover conditions, viz., no mask, helmet, rubber mask, surgeon mask, and scarf [27]. Ristea and Ionescu proposed a data augmentation approach for mask detection from speech based on training Generative Adversarial Networks (GANs) with cycle-consistency loss to translate unpaired utterances between two classes (with mask and without mask) which yielded better results than other baseline and state-of-the-art augmentation methods (i.e., a score of 74.6%) [28]. Das and Li focused on the acoustic features capturing different acoustic properties of a signal to classify speech with and without mask. They found that linear frequency cepstral coefficient (LFCC), instantaneous frequency cosine coefficients (IFCC), constant-Q cepstral coefficients (CQCC), and Mel frequency cepstral coefficient (MFCC) could lead to an average performance of 73.50% with a fusion with the state-of-the-art baselines (e.g., DeepSpectrum and auDeep features) [29].

### 1.3 Current study

In the field of forensic speaker identification, speech serves as the fundamental element for solving all cases. Not to mention that auditory, acoustic-phonetic, and (semi-)automatic speaker recognition approaches, as the prevalent paradigms in forensic speaker identification across the world [30], would be greatly impacted by the acoustic presentation of speech. Especially, the acoustic-phonetic approach mainly depends on two basic procedures: (1) rate the similarity of spectrograms between suspect recording and offender recording; (2) statistical comparison of the acoustic parameters between suspect recording and offender recording [30–32]. Therefore, the question of whether wearing face mask would exhibit a salient effect on speech production is essential to the practices of forensic speaker identification. However, no study has been conducted on the relevant topic, with an exception of Saeidi et al. which showed that wearing face cover equipment would not significantly impact the accuracy of automatic speaker recognition [27].

Although recent research has been carried out on the effects of face mask on speech production, no consensus has been reached. In addition to the inconsistent results within a particular language (i.e., English), cross-linguistic differences (e.g., results of Mandarin Chinese in [7] contradict those of English) of speech with face mask have not yet been fully investigated. Furthermore, the question of how face mask would impact forensic speaker identification remains unclear. Therefore, the current study conducted a systematical cross-linguistic analysis of the continuous speech (in both Mandarin Chinese and English versions) recorded from 30 native speakers of Mandarin Chinese (not-)wearing a surgical mask. Further, four supervised learning algorithms (i.e., Linear Discriminant Analysis, Naive Bayes classifier, Random Forest, and Support Vector Machine) were conducted to classify speech with and without mask and identify individual speakers, respectively. The current paper seeks to further understand the effects of face mask on speech production and the cross-linguistic difference in speech with face mask between Mandarin Chinese and English. Besides, this study aims to provide some instructive and practical opinions on the implementation of acoustic-phonetic and (semi-)automatic speaker recognition approaches under face mask wearing conditions in forensic speaker identification.

## 2. Methods

### 2.1 Speech materials

This research was approved by the Committee for the Protection of Human Subjects (CPHS) at the Academy of Forensic Science (Shanghai, China). Thirty native speakers of Mandarin Chinese (15 men and 15 women) were recruited for the recording session. All speakers have passed the CET-4 test (College English Test in China) and speak fluent English as their second language. The average age and height were 26.67 years (sd = 2.82) and 177.0 cm (sd = 4.33) for male speakers, and 24.13 years (sd = 2.07) and 163.27 cm (sd = 6.05) for female speakers. None of the participants had a reported history of speech or hearing disorders. All speakers received reasonable financial compensation for their participation.

Before data collection, the participants were asked to read a consent form containing the purpose of the study, confidentiality, and the rights of participants. After the participants indicated their willingness to participate, they were asked to sign the form. Informed consent was obtained from all individual participants included in the study.

Phonetically balanced texts *The North Wind and the Sun* in both Chinese and English versions (as shown in S1 Appendix) were included as the speech materials in this study. All speakers were asked to familiarize the speech materials and practice as many times as they want. Speech recording was conducted in a sound-proof booth, using a portable digital recorder Zoom H5n. Each participant read aloud *The North Wind and the Sun* text, with his/her normal speech rate, pitch, and loudness. The recording procedure was conducted for all speakers in the same sequence: twice without surgical mask in Mandarin, once with surgical mask in Mandarin, once without surgical mask in English, and once with surgical mask in English.

All speech signals were saved in the WAV format with a 48.0 kHz sampling rate at a 16-bit resolution. Altogether, 90 recordings of Mandarin (i.e., 2 [times without mask] * 30 [speaker] + 1 [time with mask] * 30 [speakers]) and 60 recordings of English (i.e., 1 [time without mask] * 30 [speakers] + 1 [time with mask] * 30 [speakers]) were collected. To make the recording order counterbalanced in the current study, the second-round recording of Mandarin speech without mask of each participant was excluded from the current study.

### 2.2 Data extraction

Mandarin and English recordings were segmented and annotated automatically at the word and phonemic levels based on Mandarin/English pre-trained acoustic models using Montreal Forced Aligner software [33] and then were manually corrected by an experienced labeler using Praat [34]. Fundamental frequency (F0) values were extracted using a short-term autocorrelation algorithm in Praat. The F0 values were checked and manually revised to correct for the “doubling” or “halving” errors in F0 tracking. The F0 values were then extracted at the ten time-normalized (i.e., equally spaced) points for each word, measured in semitone (st) with a reference of 100 Hz [i.e., st = 12*log2(F0 value/100)]. Also, duration and intensity were measured for each word. Because of the different syllable structures between Mandarin Chinese and English (i.e., monosyllabic vs. multisyllabic), speech rate (syllables/second) was then calculated for further analysis. Besides, voice quality measures, viz., jitter, shimmer, harmonic-to-noise ratio (HNR), and H1-H2, were extracted from the vocalic segments within each word using Praat.

### 2.3 Statistical analysis

All acoustic parameters, including F0, speech rate, intensity, and voice quality measures were statistically analyzed using R [35]. Linear-mixed effect model [36] was conducted to analyze statistical differences of the above parameters between with and without face mask conditions across languages, with the acoustic parameters as dependent variables, and mask (i.e., with vs. without), language (i.e., Mandarin Chinese vs. English), and gender (i.e., male vs. female) as independent variables. The random intercepts for speaker as well as the random slopes for mask and language by speaker were included in the model to support the maximal random effect structure design [37]. The significance of the random slopes was then checked using likelihood ratio test and showed that all slopes were significant in model fitting. Therefore, all random slopes were included in the model (Model<-lmer (acoustic parameter ~ mask*gender*language+(1+mask*language|speaker), data = data)). Tukey HSD post hoc tests were then conducted to make pairwise comparisons [38].

## 3. Results of acoustic analysis

**Fig 1:**
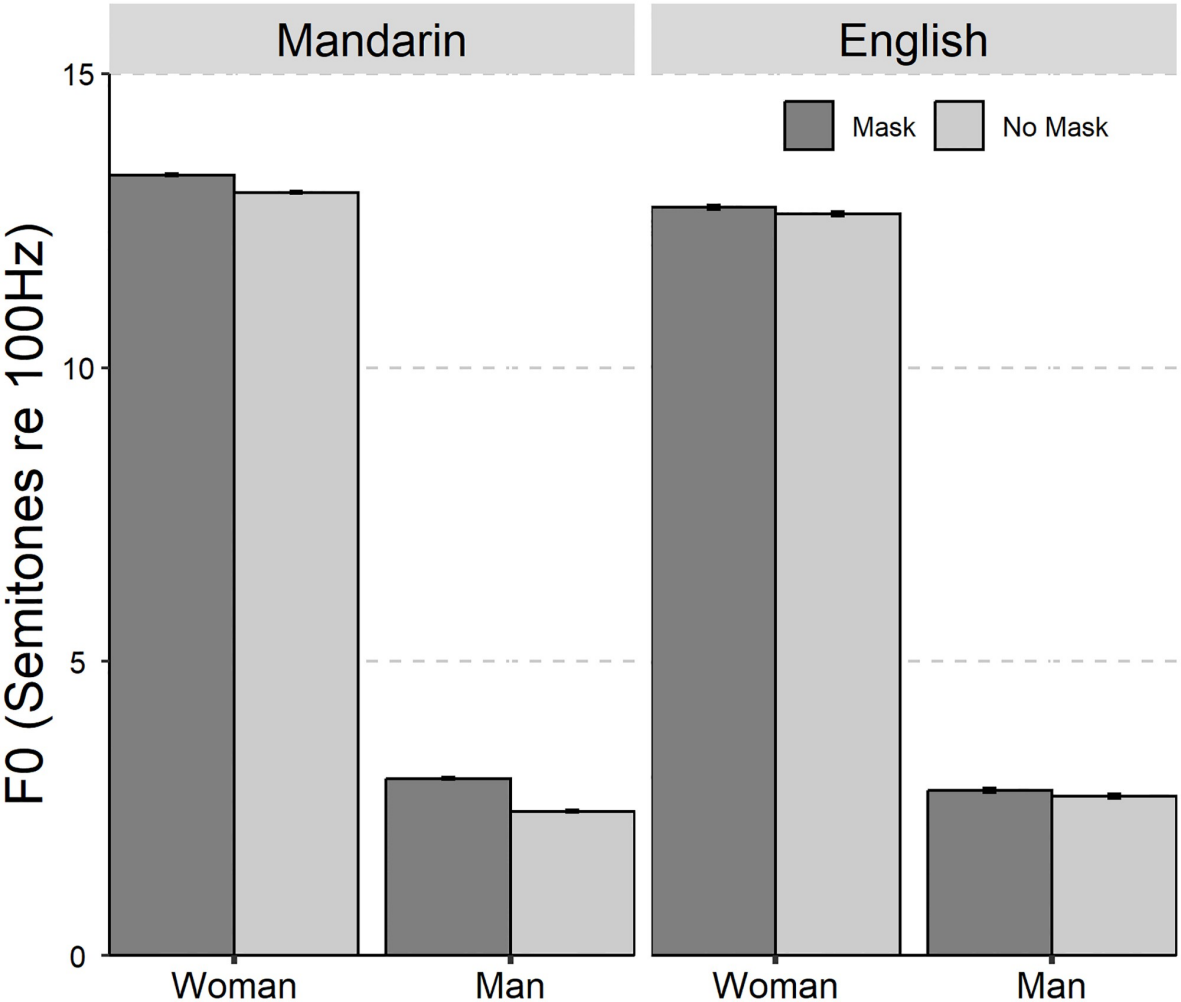
The bar plot (with standard error) of the fundamental frequency (in semitones with a reference of 100 Hz) of Mandarin Chinese and English with and without mask across gender (female and male).

**Fig 2:**
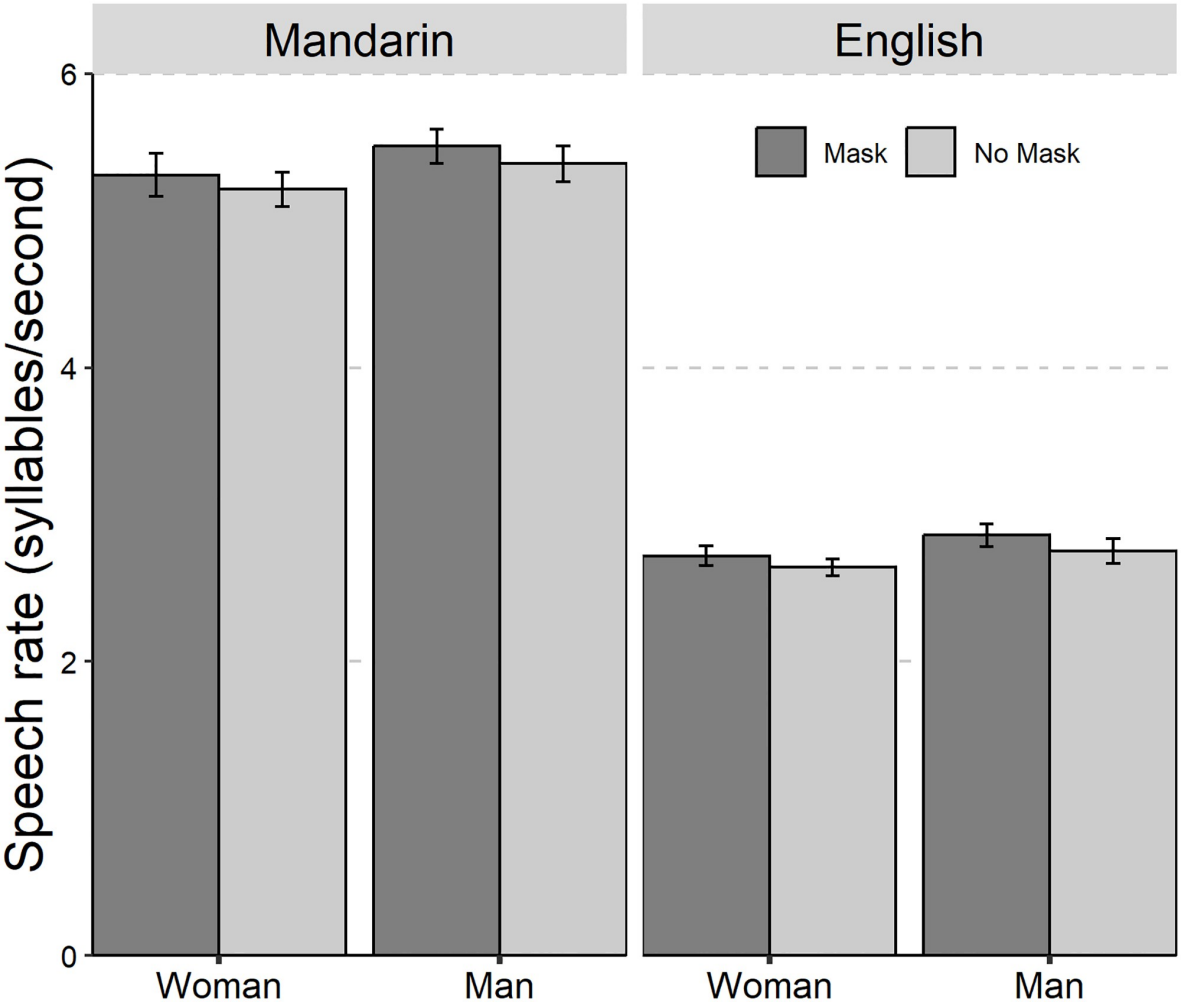
The bar plot (with standard error) of the speech rate (syllables/second) of Mandarin Chinese and English with and without mask across gender (female and male).

**Fig 3:**
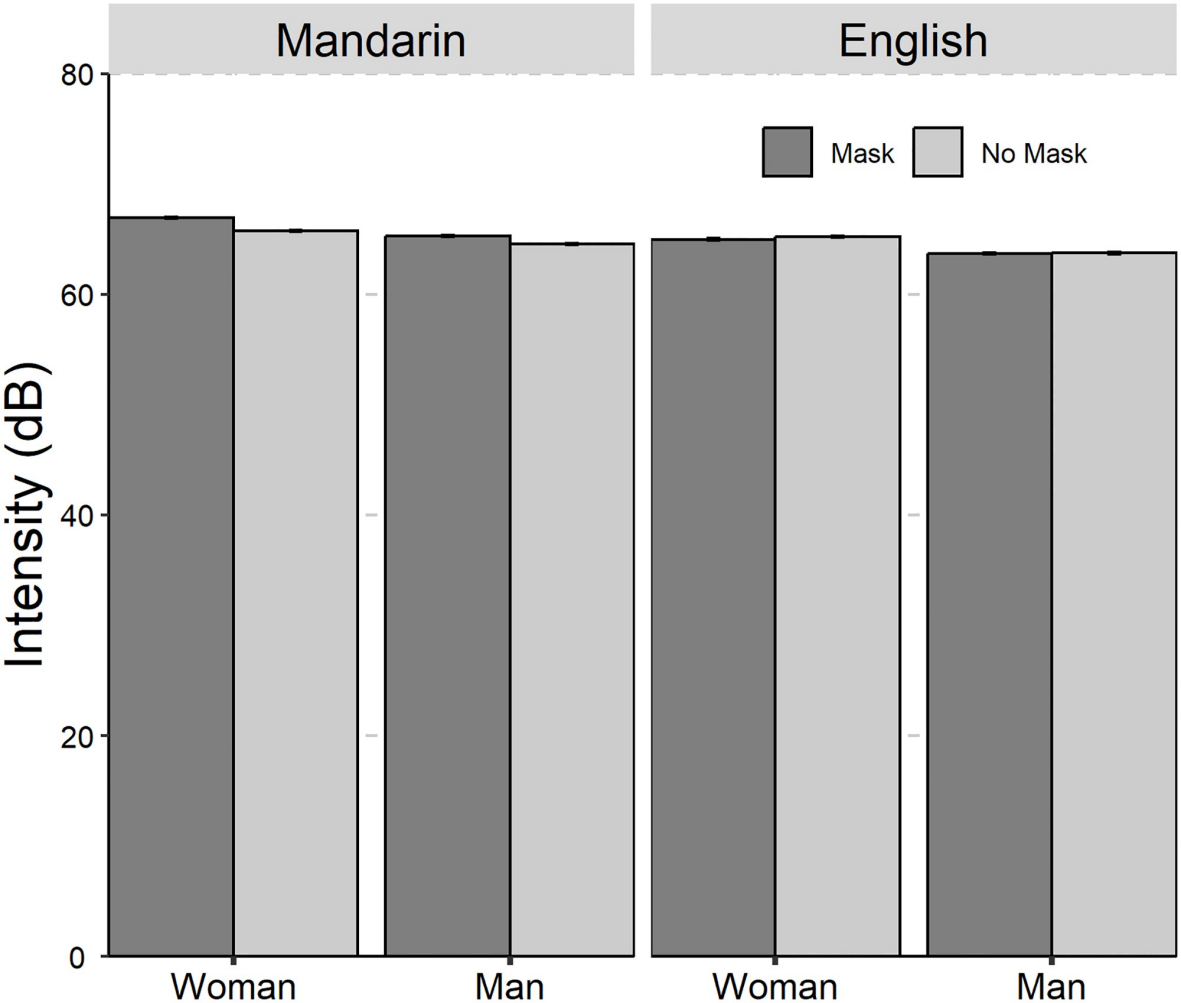
The bar plot (with standard error) of the intensity (in dB) of Mandarin Chinese and English with and without mask across gender (female and male).

**Fig 4:**
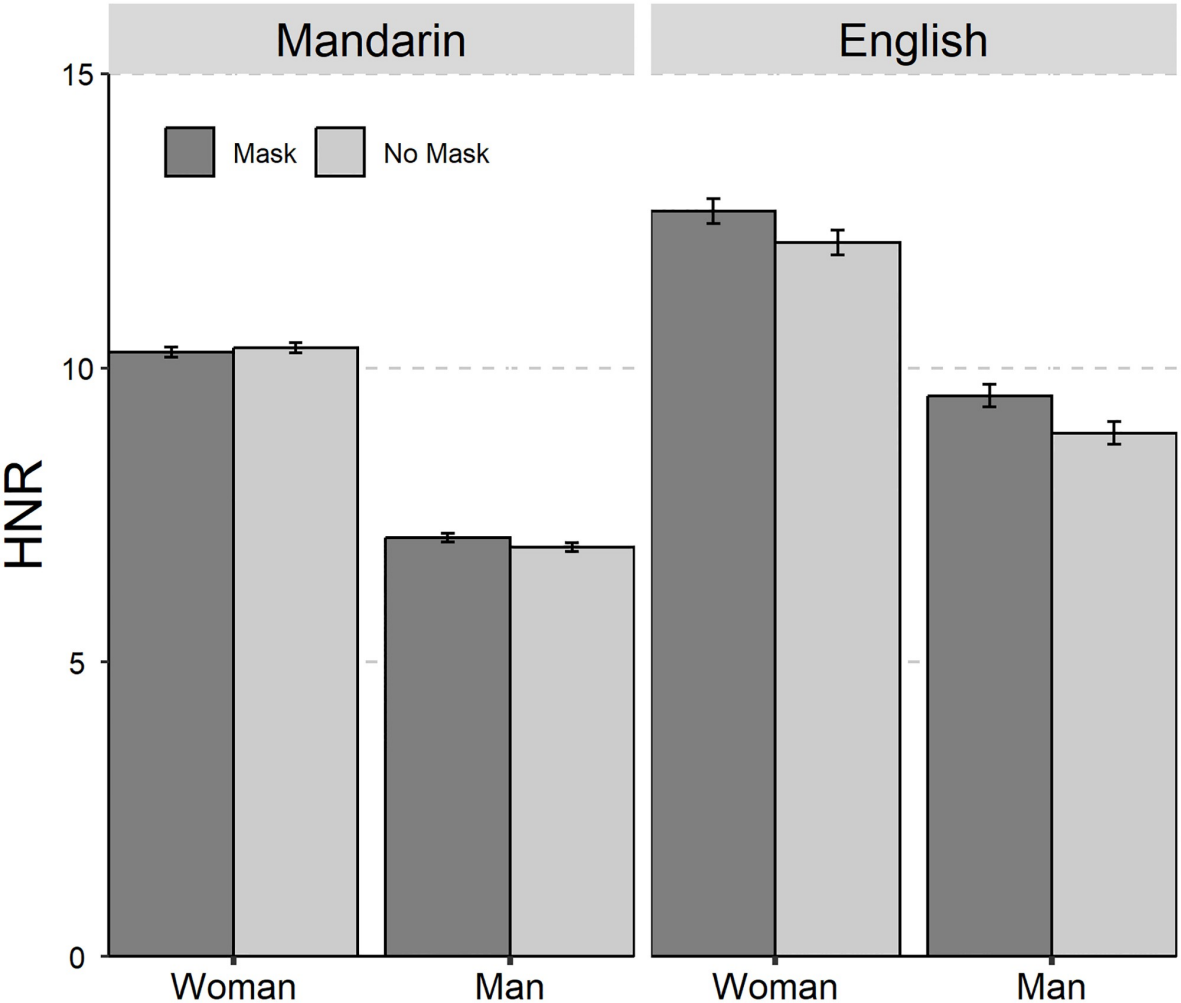
The bar plot (with standard error) of the harmonic-noise-ratio (HNR in dB) of Mandarin Chinese and English with and without mask across gender (female and male).

**Fig 5:**
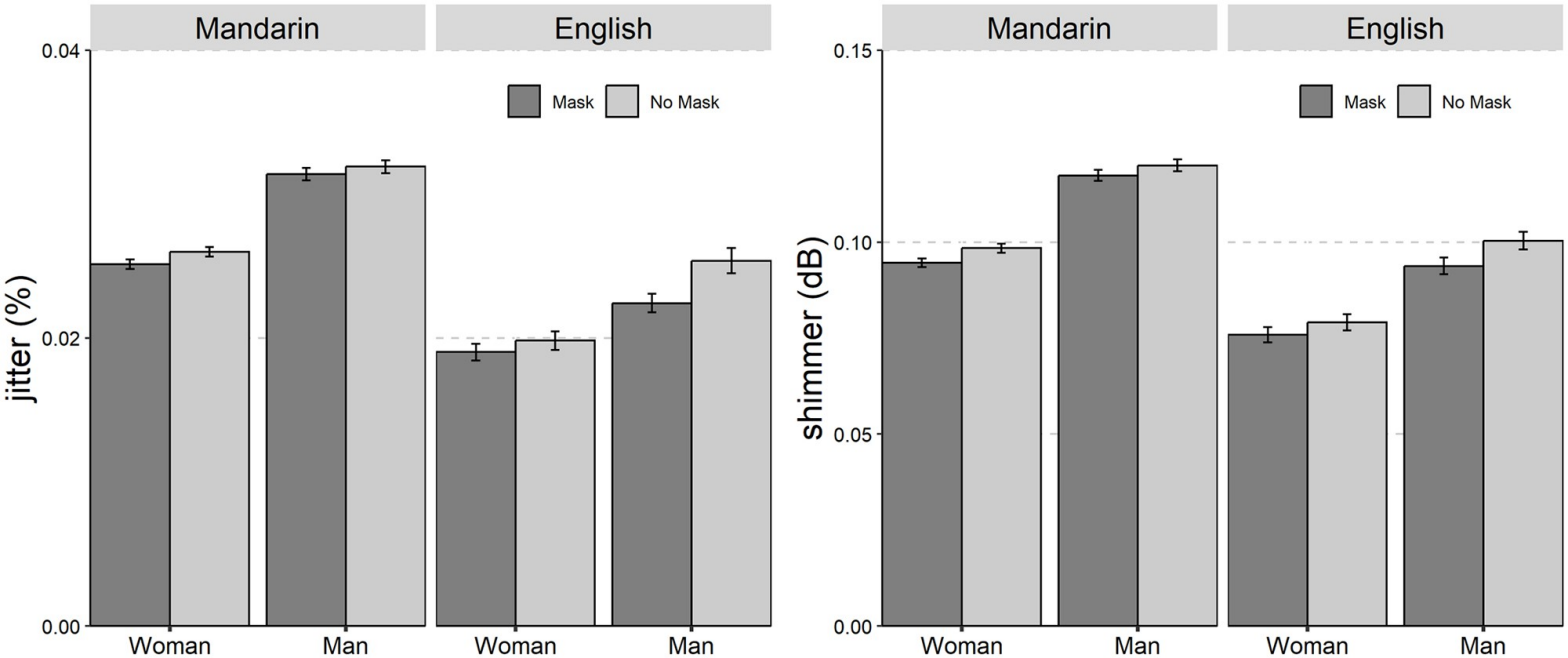
The bar plot (with standard error) of the jitter (in %) and shimmer (in dB) of Mandarin Chinese and English with and without mask across gender (female and male).

**Fig 6:**
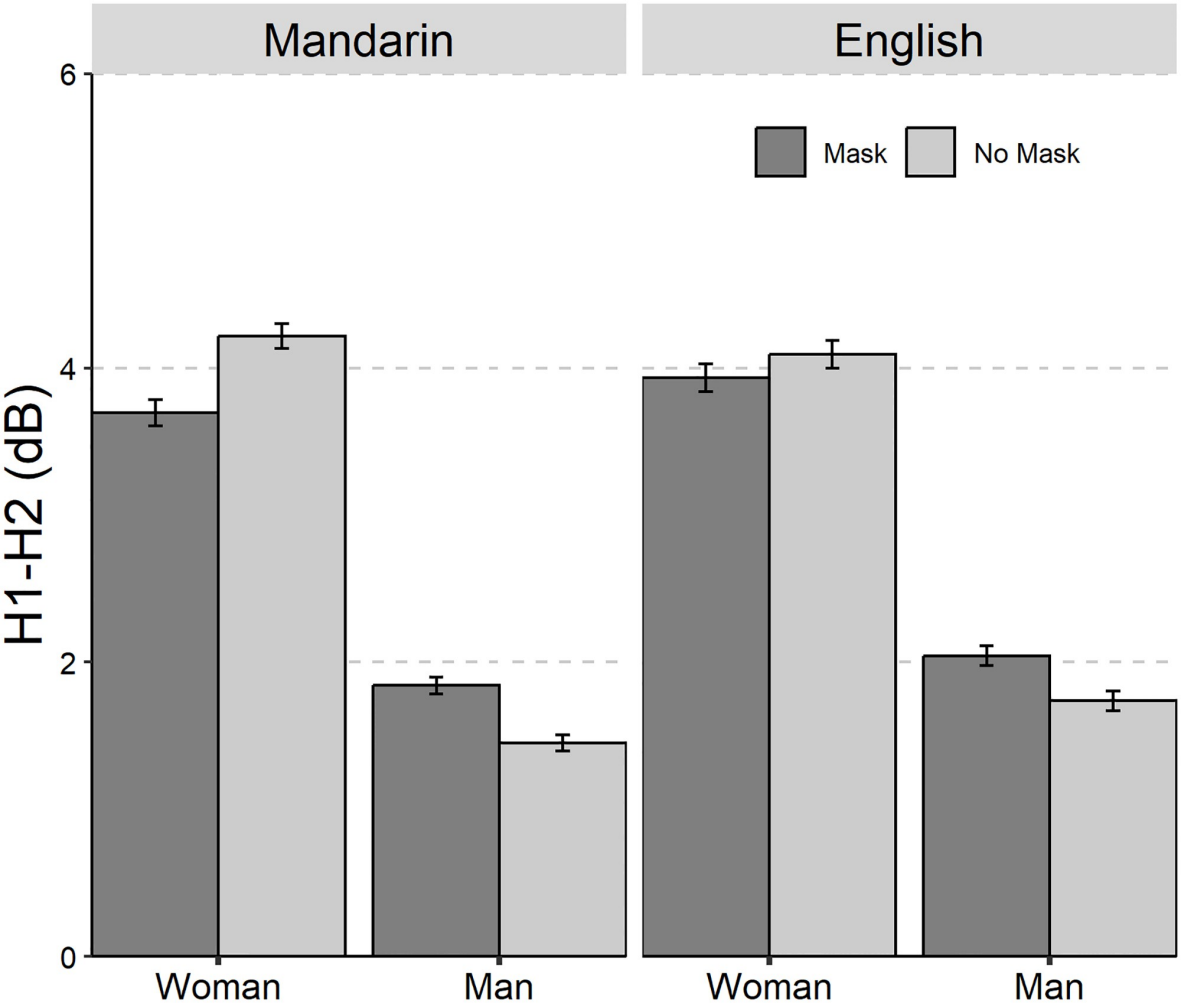
The Bar plot (with standard error) of the H1-H2 (in dB) of Mandarin Chinese and English with and without mask across gender (female and male).

The average values and standard deviations are presented in Table 1. The results of the linear mixed-effect models on the seven acoustic parameters are summarized in Table 2. The seven acoustic parameters of Mandarin Chinese and English with and without mask across gender was shown in Figs 1 to 6.

**Table 1:** Average values (standard deviation) of the seven acoustic parameters with and without mask in Mandarin Chinese and English.

	Mandarin Chinese				English			
	Male		Female		Male		Female	
	No-mask	Mask	No-mask	Mask	No-mask	Mask	No-mask	Mask
**F0**	2.71	2.81	12.61	12.73	2.51	3.01	13.06	13.28
	(2.34)	(2.33)	(2.51)	(2.38)	(3.02)	(3.14)	(3.1)	(3.24)
**Speech**	5.39	5.51	5.21	5.31	2.75	2.86	2.64	2.72
**rate**	(0.47)	(0.46)	(0.46)	(0.56)	(0.32)	(0.3)	(0.23)	(0.26)
**Intensity**	64.87	65.3	66.13	66.95	63.75	63.69	65.22	64.99
	(9.35)	(9.74)	(8.56)	(8.75)	(10.65)	(10.83)	(10)	(10.07)
**HNR**	6.84	7.12	10.1	10.27	8.89	9.53	12.13	12.66
	(3.56)	(3.61)	(4.3)	(4.3)	(3.76)	(3.76)	(4.09)	(4.08)
**Jitter**	3.18	3.14	2.64	2.51	2.54	2.24	1.98	1.9
	(2.18)	(2.17)	(1.75)	(1.65)	(1.71)	(1.25)	(1.26)	(1.12)
**Shimmer**	12.19	11.74	10.06	9.46	10.04	9.38	7.91	7.59
	(7.77)	(7.26)	(5.91)	(5.58)	(4.45)	(4.11)	(4.05)	(3.79)
**H1-H2**	1.46	1.84	4.12	3.69	1.74	2.04	4.09	3.93
	(6.25)	(6.29)	(9.46)	(9.99)	(6.32)	(6.1)	(8.75)	(8.7)

**Table 2:** Results of the linear mixed-effect models on the seven acoustic parameters with mask (with vs. without), gender (female vs. male), and language (Mandarin Chinese vs. English) as independent variables. The asterisks indicate the levels of statistical significance: * *p* < 0.05; ** *p* < 0.01; *** *p* < 0.001.

**Factor**	**Acoustic parameter**							
	**F0**		**Speech rate**		**Intensity**		**HNR**	
	***F***	***p***	***F***	***p***	***F***	***p***	***F***	***p***
**Mask**	20.29	***	3.37	0.07	4.46	*	5.81	*
**Gender**	255.23	***	2.00	0.17	1.41	0.26	159.65	***
**Language**	1.91	0.18	2254.35	***	13.36	***	145.11	***
**Mask×Gender**	1.07	0.31	0.05	0.82	0.14	0.71	0.40	0.53
**Mask×Language**	9.83	**	0.02	0.89	16.27	***	3.49	0.07
**Gender×Language**	2.44	0.13	0.29	0.59	0.00	0.95	0.05	0.82
**Mask×Gender×Language**	1.85	0.18	0.00	0.97	1.31	0.26	0.04	0.84
	**Jitter**		**shimmer**		**H1-H2**			
	***F***	***p***	***F***	***p***	***F***	***p***		
**Mask**	6.05	*	4.22	*	0.00	0.97		
**Gender**	31.69	***	24.97	***	10.68	**		
**Language**	93.53	***	86.21	***	0.85	0.36		
**Mask×Gender**	0.75	0.39	0.07	0.79	9.85	**		
**Mask×Language**	1.09	0.30	0.17	0.68	0.64	0.43		
**Gender×Language**	1.35	0.25	0.41	0.52	0.33	0.57		
**Mask×Gender×Language**	1.18	0.28	0.28	0.60	1.64	0.21		

The main effect of “Mask” and “Gender”, and the two-way interaction effect ***of*** “Mask×Language” were significant for F0. If there are related higher-order interactions, the main effect or lower-order interaction will not be discussed. The Tukey HSD *post hoc* test was then conducted on the two-way interaction effect of “Mask×Language”. The results showed that F0 is higher in the mask speech than in the no mask speech for Mandarin Chinese, while no significant effect was found for English (MC: mask-no mask, *β* = 0.43 st, *SE* = 0.06, *z* = 7.20, *p* < 0.001; E: mask-no mask, *β* = 0.11 st, *SE* = 0.09, *z* = 1.16, *p* = 0.25).

As for Speech rate, only a significant effect of “Language” was found. The *post hoc* test was not performed since no significance of the interested factor (i.e., “Mask”) was found.

The significant main effect of “Mask” and “Language”, and the two-way interaction effect of “Mask×Language” were found for intensity. The *post hoc* test on the two-way interaction effect of “Mask×Language” showed that speech with face mask exhibited higher intensity than speech without face mask for Mandarin Chinese (mask-no mask: *β* = 0.97 dB, *SE* = 0.26, *z* = 3.70, *p* < 0.001), while no significant effect was found for English (mask-no mask: *β* = -0.15 dB, *SE* = 0.21, *z* = -0.68, *p* = 0.50).

The main effects of “Mask”, “Gender”, and “Language” were significant for HNR. No significant interaction effect was found for HNR. The Tukey-HSD *post hoc* test on the main effect of “Mask” showed that speech wearing face mask exhibited higher HNR than speech without wearing face mask (mask-no mask: *β* = 0.31 dB, *SE* = 0.13, *z* = 2.41, *p* = 0.02).

The significant main effects of “Mask”, “Gender”, and “Language” were found for both jitter and shimmer measurements. The results of Tukey-HSD *post hoc* test showed that speech with face mask was produced with lower jitter (mask-no mask: *β* = -0.13%, *SE* = 0.05, *z* = -2.46, *p* = 0.01) and shimmer (mask-no mask: *β* = -0.41 dB, *SE* = 0.20, *z* = -2.06, *p* = 0.04) than speech without face mask.

The significant main effect of “Gender” and the two-way interaction effect of “Mask×Gender” were found for H1-H2. Tueky-HSD *post hoc* test on the two-way interaction effect of “Mask×Gender” showed that both female speakers produced lower H1-H2 in mask speech than in no mask speech (mask-no mask: *β* = -0.34 dB, *SE* = 0.16, *z* = -2.19, *p* = 0.03), while the opposite pattern was observed for male speakers (mask-no mask: *β* = 0.35 dB, *SE* = 0.16, *z* = 2.25, *p* = 0.03).

## 4. Classification analyses based on four supervised learning algorithms

Four supervised learning algorithms, viz., linear discriminant analysis (LDA), naïve Bayes classifier (NBC), random forest (RF), and support vector machine (SVM) were performed to classify the speech with and without face mask using R packages [39–42]. All seven acoustic parameters were included as the predictor variables. Given that a cross-linguistic difference was observed, separate classification analyses were conducted on all speech, Mandarin speech, and English speech, respectively. For NBC, RF, and SVM algorithms, data were divided into train and test sets in a ratio of 7:3. The optimized parameters and classification accuracies of each algorithm were presented in Table 3.

**Table 3:** Optimized parameters and accuracies of the automatic classification analyses on speech with and without face mask across language based on the four supervised learning algorithms [i.e., linear discriminant analysis (LDA), naïve bayes classifier (NBC), random forest (RF), and support vector machine (SVM)]. All seven acoustic parameters were included as the predictor variables.

		LDA	NBC	RF	SVM
All data	Optimized parameters	1^st^ canonical discriminant function wilks’ λ = 0.96 χ^2^ (7) = 5.28	Laplace = 0 usekernal = T	ntree = 1000 mtry = 2	vectors = 77 cost = 1
	Accuracy (%)	54.2	44.44	33.33	44.44
	[95%CI]	[52.77, 55.63]	[43.27, 45.61]	[32.45, 34.21]	[43.27, 45.61]
Mandarin Chinese	Optimized parameters	1^st^ canonical discriminant function wilks’ λ = 0.93 χ^2^ (7) = 3.98	Laplace = 0 usekernal = T	ntree = 1000 mtry = 3	vectors = 33 cost = 1
	Accuracy (%)	68.3	30.43	50	22.22
	[95%CI]	[66.50, 70.10]	[13.21, 52.92]	[48.68, 51.32]	[21.64, 22.81]
English	Optimized parameters	1^st^ canonical discriminant function wilks’ λ = 0.88 χ^2^ (7) = 6.91	Laplace = 0 usekernal = T	ntree = 1000 mtry = 2	vectors = 28 cost = 1
	Accuracy (%)	66.7	45.45	22.22	22.22
	[95%CI]	[64.95, 68.46]	[24.39, 67.79]	[21.64, 22.81]	[21.64, 22.81]

**Fig 7:**
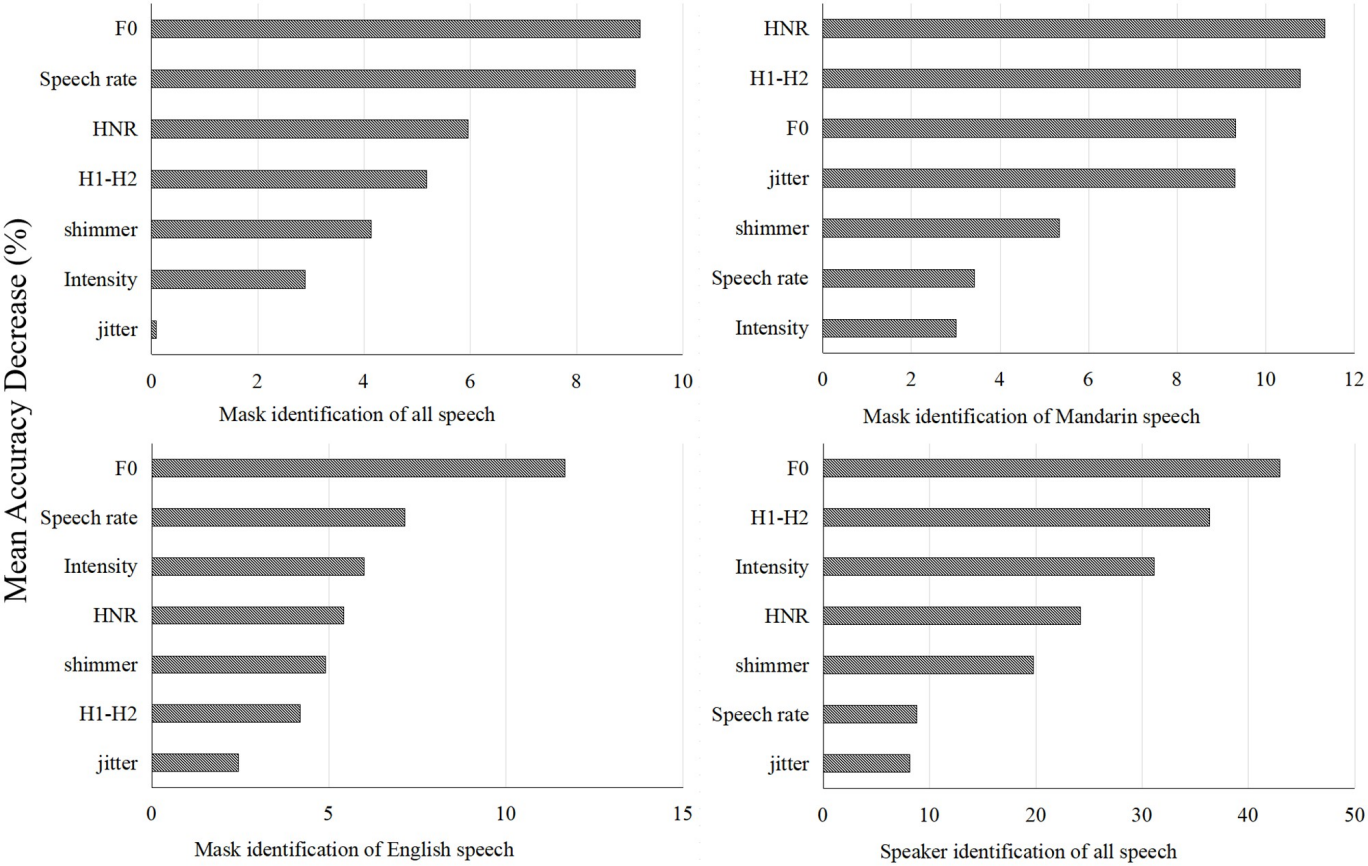
The significance of the seven acoustic parameters in the random forest models for mask speech identification (data: All speech, Mandarin speech, and English speech) and individual speaker identification (data: All speech mixed by mask and no mask speech).

According to the results in Table 3, the accuracies of identification on mask and no mask speech ranged from 22.22% to 68.3%. LDA, followed by NBC, showed better accuracies on mask/no mask speech classification than RF and SVM. However, only LDA algorithm showed accuracies higher than 50% (i.e., chance level). The significance (i.e., mean accuracy decrease) of the seven acoustic parameters in the random forest models for mask speech identification (data: all speech, Mandarin speech, and English speech) were shown in Fig 7. For Mandarin Chinese, aperiodicity parameters (i.e., F0, HNR, and H1-H2) played more important roles in the classification of mask and no mask speech, while F0, speech rate, and intensity contributed more to the accuracy of English mask/no mask speech classification.

To investigate the accuracy of individual speaker identification under the condition of face mask wearing, the same procedures of classification analyses were conducted. All speech data mixed by mask and no mask speech were included in the classification analyses and split into train and test datasets in a ratio of 7:3. According to Table 4, the accuracies of speaker identification based on the four supervised learning algorithms ranged from 40% to 89.2%. LDA, followed by RF, showed better accuracies on speaker identification than NBC and SVM. As shown in Fig 7, F0, H1-H2, and intensity played more important roles in speaker identification.

**Table 4:** Optimized parameters and accuracies of the automatic speaker identification on all speech data based on the four supervised learning algorithms [i.e., linear discriminant analysis (LDA), naïve bayes classifier (NBC), random forest (RF), and support vector machine (SVM)]. All seven acoustic parameters were included as the predictor variables.

	LDA	NBC	RF	SVM
Optimized parameters	7 canonical discriminant functions wilks’ λ = 0.97 χ^2^ (23) = 3.02	Laplace = 0 usekernal = T	ntree = 1000 mtry = 2	vectors = 88 cost = 1
Accuracy (%)	89.2	40	72.22	53.33
[95%CI]	[86.85, 91.55]	[25.70, 55.67]	[70.32, 74.12]	[51.93, 54.73]

## 5. Discussion

The current study aims to investigate (1) the effect of face mask on Mandarin and English speech production, (2) the accuracy of mask/no mask speech classification based on acoustic parameters, and (3) the effect of face mask on automatic speaker recognition. Taking surgical mask as an example, the results of acoustic analyses showed a cross-linguistic difference in the mask speech production: mask speech exhibited higher F0, intensity, and HNR, and lower jitter and shimmer than no mask speech for Mandarin Chinese, whereas higher HNR, and lower jitter and shimmer were observed for English mask speech. Besides, a cross-gender difference was also observed that female speakers exhibited a lower H1-H2 and male speakers exhibited a higher H1-H2 in mask speech production. The results of the automatic identification of mask/no mask speech and automatic speaker recognition based on the four supervised learning algorithms further revealed that, at least for surgical mask, undesirable performances (i.e., lower than 50%) in classifying the speech with and without face mask and highly-variable accuracies in identifying individual speakers (e.g., ranging from 40% to 89.2%) were achieved based on the acoustic parameters.

### 5.1 Effect of face mask on speech production

As reviewed in section 1.1, previous studies based on a sustained /a/ revealed that different results were found between English and Mandarin Chinese speakers (e.g., no significant effect of face mask for English in [3] vs. the significant effect of face mask for Mandarin in [7]). It is therefore reasonable to hypothesize that, in spite of speech materials, face mask might exhibit different effects on speech production across languages. The findings of the current study support the above hypothesis that face mask seems to exhibit more significant effects on Mandarin Chinese than on English. In line with the opinions of previous studies [14, 15], our findings implied that the speakers tend to conduct acoustic adjustments to improve their speech intelligibility when wearing surgical mask. However, a cross-linguistic difference in the strategies to compensate for speech intelligibility was observed that Mandarin speech was produced with higher F0, intensity (i.e., louder), and HNR (i.e., higher ratio of the harmonic component than the noise component, which is associated with a clearer voice. [43]), while English was produced with higher HNR.

Surprisingly, the voice quality measurements (i.e., HNR, jitter, shimmer, and H1-H2) showed a consistent pattern in mask speech production between Mandarin Chinese and English. Therefore, a clearer voice and a smaller perturbation (i.e., lower jitter and shimmer) in mask speech production might be a general pattern across languages. However, some scholars have argued that KN95 mask demonstrated a greater effect on speech acoustics than surgical mask [13]. Therefore, with the missing of other face mask types (e.g., N95 respirator, cloth mask) in the current study, caution must be applied, as the findings might not be applicable to all face masks. More comprehensive studies on other face masks and languages are then recommended to draw a more general conclusion about the effect of face mask on speech production.

Although significant changes have been observed in the acoustic presentation of mask speech, the acoustic parameters showed undesirable performances (i.e., the accuracies were lower than 50%) on mask and no mask speech classification based on the four supervised learning algorithms. Some scholars have reported that other features (e.g., constant-Q cepstral coefficients, Mel frequency cepstral coefficient, etc.) might contribute to a relatively good accuracy (i.e., around 70%) in identifying mask speech [29]. Hence, other methods of feature extraction are necessary for future studies to seek a better resolution of mask speech classification. Besides, according to the results of automatic speaker identification, a further comparison among the four supervised learning algorithms shows an ascending order of accuracies, viz., NBC (i.e., 40%) < SVM (i.e., 53.33%) < RF (i.e., 72.22%) < LDA (i.e., 89.2%). It is possible, therefore, that the surgical mask would impact the general performance of the accuracy of automatic speaker recognition to some extent.

### 5.2 Does face mask impact forensic speaker identification

**Fig 8:**
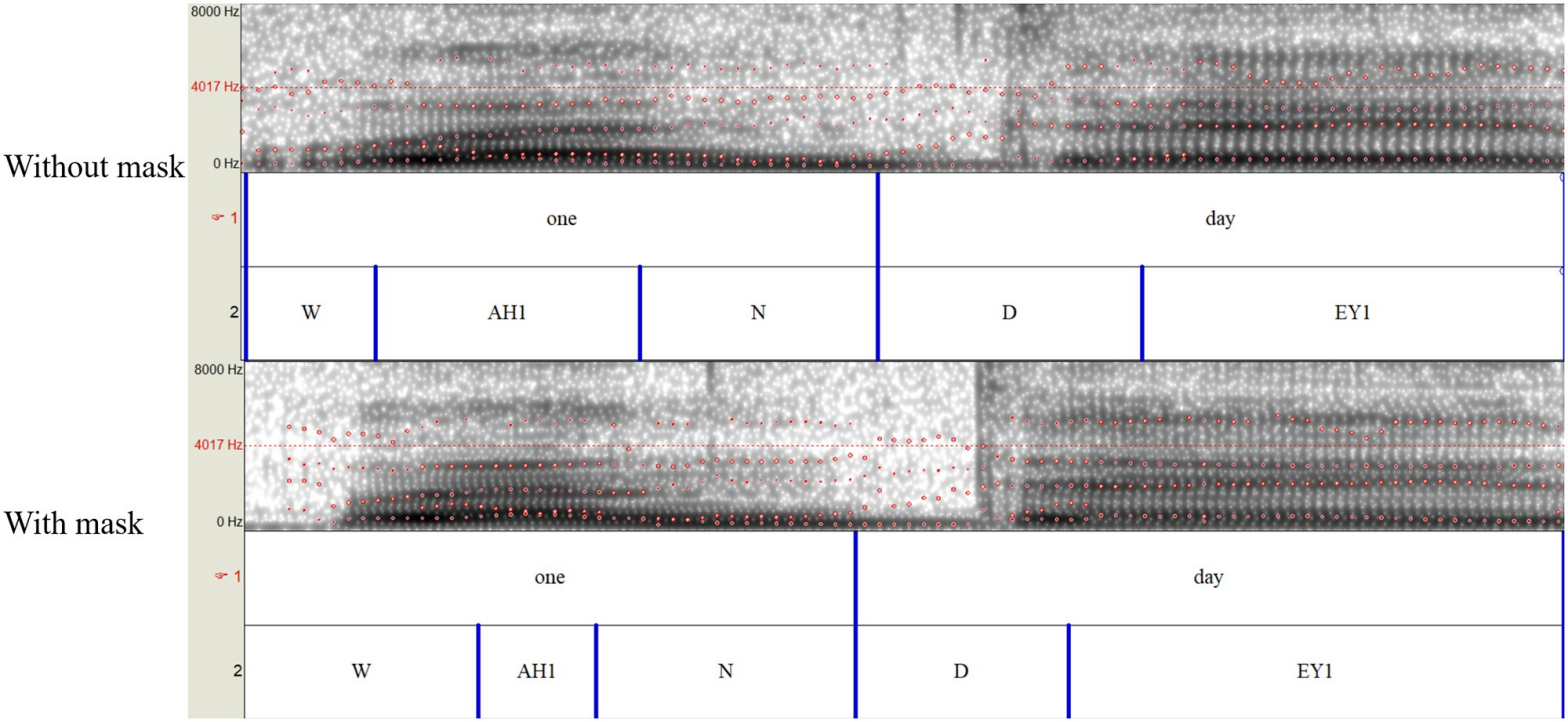
An example of spectrogram on the same segment (“one day” in English) of no mask speech (above) and mask speech (below). The speech was annotated in two layers, viz., word (i.e., first layer) and phoneme (i.e., second layer; in a ARPABET phone set). The red dots indicate the first four formants of speech.

Another important question of the current paper is whether wearing a face mask will impact the implementation of forensic speaker identification. An example of the spectrogram on the same segment of mask and no mask speech (Speaker_01) was presented in Fig 8. The presentations of the spectrogram show comparability between speech with and without wearing surgical mask. However, the findings of the current study show significant effects of surgical mask on the acoustic presentations of mask speech, which indicate that caution should be required in the acoustic-phonetic approach of forensic speaker identification under face mask wearing condition. Besides, it should be noted that the acoustic changes in mask speech (Mandarin: higher F0, intensity, and HNR; English: higher HNR) might lead to a perceptual variation (e.g., clearer voice with more speech effort. [43]), which also required particular attention in the auditory paradigm of forensic speaker identification.

Further, based on the acoustic parameters, highly-variable accuracies (i.e., ranging from 40% to 89.2%) of speaker identification were achieved using the four supervised learning algorithms. It can thus be suggested that, at least for surgical mask, the automatic speaker recognition approaches of forensic speaker identification would be impacted by face mask to some extent. In future studies, however, other types of face mask would certainly be necessary to be investigated to draw a firm and comprehensive conclusion.

Several limitations of the present study should also be noted. In the first place, as the current study has shown that surgical mask exhibits significant effects on speech production, the acoustic presentations of speech across different types of face masks (e.g., N95 respirator, cloth mask, and surgical mask) will need to be undertaken. Secondly, the cross-linguistic/-gender differences revealed in this paper not only suggest that the mask speech might show language-dependent acoustic patterns but also indicate that speakers could have different speech strategies (e.g., more effort vs. less effort) when wearing a face mask. Future studies, which take other languages and individual differences into account, are therefore recommended. Last but not least, more learning algorithms (e.g., Feed Forward Neural Network, [44]) and feature extraction techniques (e.g., MFCCs, i-vector, etc.) should be examined to seek better resolutions of the identifications on mask speech and individual speakers.

## 6. Conclusion

The current study preliminary revealed that speakers tend to conduct acoustic adjustments to improve their speech intelligibility when wearing surgical mask. However, a cross-linguistic difference in speech strategies to compensate for intelligibility was observed that Mandarin speech was produced with higher F0, intensity (i.e., louder), and HNR (indicates clearer voice), while English was produced with higher HNR. Further, based on the four supervised learning algorithms, the accuracies of classification on mask and no mask speech were undesirable, while highly-variable accuracies of speaker identification were achieved under a face mask wearing condition. In general, therefore, it seems wearing surgical mask would impact both acoustic-phonetic and automatic speaker recognition approaches to some extent, thus suggesting particular cautions in the real-case practice of forensic speaker identification.

## Supporting information

S1 File Mandarin speech of speaker 1–15. This is the Mandarin speech materials from speakers 1–15 analyzed in the current study.
(RAR)

S2 File Mandarin speech of speaker 16–30. This is the Mandarin speech materials from speakers 16–30 analyzed in the current study.
(RAR)

S3 File English speech of speaker 1–15. This is the English speech materials from speakers 1–15 analyzed in the current study.
(RAR)

S4 File English speech of speaker 16–30. This is the English speech materials from speakers 16–30 analyzed in the current study.
(RAR)

S1 Appendix Speech materials in Chinese and English versions. (DOCX)
