# Supplementary material for: The effects of face mask on speech production and its implication for forensic speaker identification-A cross-linguistic study
Source: PLoS One. 2023 Mar 30;18(3):e0283724. doi: 10.1371/journal.pone.0283724 (PMC10062611; doi:10.1371/journal.pone.0283724)
Supplement: S1 Appendix — (DOCX) [file pone.0283724.s005.docx]

**Appendix 1:** **Speech materials in Chinese and English versions**

a) Chinese version (in pinyin):

you3 yi4 tian1, bei3 feng1 gen1 tai4 yang2 zai4 na4 li3 zheng1 lun4 shei2 de5 ben3 shi4 geng4 da4. shuo1 zhe5 shuo1 zhe5, lai2 le5 yi2 ge4 zou3 lu4 de5 ren2. shen1 shang5 chuan1 le5 yi2 jian4 hou4 pao2 zi5. ta1 men5 lia3 jiu4 shang1 liang5 hao3 le5. shuo1 shei2 nai4 jiao4 zhe4 ge5 zou3 lu4 de5 ren2 ba3 ta1 de5 pao2 zi5 tuo1 xia4 lai2, jiu4 suan4 shei2 de5 ben3 ling3 da4. yu2 shi4 bei3 feng1 jiu4 mao3 zu2 le5 jiner4, pin1 ming4 de5 chui1 ya1 chui1 ya1. ke3 shi4, ta1 chui1 de5 yue4 li4 hai5, na4 ge5 ren2 jiu4 ba3 ta1 de5 pao2 zi5 guo3 de5 yue4 jin3. dao4 le5 zui4 hou4, bei3 feng1 mei2 zhe2 le5, zhi3 hao3 jiu4 suan4 le5. yi2 huier4, tai4 yang2 chu1 lai2 yi2 shai4, na4 ge5 ren2 ma3 shang4 jiu4 ba3 ta1 de5 pao2 zi5 tuo1 le5 xia4 lai2. suo3 yi3, bei3 feng1 bu4 de2 bu4 cheng2 ren4, hai2 shi5 tai4 yang2 bi3 ta1 de5 ben3 ling3 da4.

b) English version:

One day, the North Wind and the Sun were disputing which was the stronger, when a traveler came along wrapped in a warm cloak. They agreed that the one who first succeeded in making the traveler take his cloak off should be considered stronger than the other. Then the North Wind blew as hard as he could, but the more he blew the more closely did the traveler fold his cloak around him; and at last the North Wind gave up the attempt. Then the Sun shined out warmly, and immediately the traveler took off his cloak. And so the North Wind was obliged to confess that the Sun was the stronger of the two.
